# Common miR-590 Variant rs6971711 Present Only in African Americans Reduces miR-590 Biogenesis

**DOI:** 10.1371/journal.pone.0156065

**Published:** 2016-05-19

**Authors:** Xiaoping Lin, Steven Steinberg, Suresh K. Kandasamy, Junaid Afzal, Blaid Mbiyangandu, Susan E. Liao, Yufan Guan, Celia P. Corona-Villalobos, Scot J. Matkovich, Neal Epstein, Dotti Tripodi, Zhaoxia Huo, Garry Cutting, Theodore P. Abraham, Ryuya Fukunaga, M. Roselle Abraham

**Affiliations:** 1 Hypertrophic Cardiomyopathy Center of Excellence, Department of Medicine, Johns Hopkins University School of Medicine, Baltimore, MD, United States of America; 2 Department of Cardiology, the Second Affiliated Hospital, School of Medicine, Zhejiang University, Hangzhou, China; 3 Institute of Genetic Medicine, Johns Hopkins University School of Medicine, Baltimore, MD, United States of America; 4 Department of Biological Chemistry, Johns Hopkins School of Medicine, Baltimore, MD, United States of America; 5 Center for Pharmacogenomics, Department of Internal Medicine, Washington University School of Medicine, St. Louis, MO, United States of America; 6 Laboratory of Molecular Cardiology, National Heart, Lung, and Blood Institute, National Institutes of Health, Bethesda, MD, United States of America; 7 Clinical Research Center, the First Affiliated Hospital, School of Medicine, Zhejiang University, Hangzhou, China; Harbin Medical University, CHINA

## Abstract

MicroRNAs (miRNAs) are recognized as important regulators of cardiac development, hypertrophy and fibrosis. Recent studies have demonstrated that genetic variations which cause alterations in miRNA:target interactions can lead to disease. We hypothesized that genetic variations in miRNAs that regulate cardiac hypertrophy/fibrosis might be involved in generation of the cardiac phenotype in patients diagnosed with hypertrophic cardiomyopathy (HCM). To investigate this question, we Sanger sequenced 18 miRNA genes previously implicated in myocyte hypertrophy/fibrosis and apoptosis, using genomic DNA isolated from the leukocytes of 199 HCM patients. We identified a single nucleotide polymorphism (rs6971711, C57T SNP) at the 17th position of mature miR-590-3p (= 57th position of pre-miR-590) that is common in individuals of African ancestry. SNP frequency was higher in African American HCM patients (n = 55) than ethnically-matched controls (n = 100), but the difference was not statistically significant (8.2% vs. 6.5%; p = 0.5). Using a cell culture system, we discovered that presence of this SNP resulted in markedly lower levels of mature miR-590-5p (39 ± 16%, p<0.003) and miR-590-3p (20 ± 2%, p<0.003), when compared with wild-type (WT) miR-590, without affecting levels of pri-miR-590 and pre-miR-590. Consistent with this finding, the SNP resulted in reduced target suppression when compared to WT miR-590 (71% suppression by WT vs 60% suppression by SNP, p<0.03). Since miR-590 can regulate TGF-β, Activin A and Akt signaling, SNP-induced reduction in miR-590 biogenesis could influence cardiac phenotype by de-repression of these signaling pathways. Since the SNP is only present in African Americans, population studies in this patient population would be valuable to investigate effects of this SNP on myocyte function and cardiac physiology.

## Introduction

MicroRNAs (miRNA) play important regulatory roles in cardiac development and pathology via post-transcriptional gene silencing[1–3]. Base-pairing between the highly conserved, 5′ proximal seed region (residues 2–8) of miRNA and the 3′-UTR (un-translated region) of target mRNA is important for miRNA: mRNA binding and target gene silencing[4, 5]. Additionally, sequences outside the seed region can also impact target suppression [6–8]. Recent studies have shown that genetic variations in miRNA genes can predispose to disease [9–13]. However, it is unknown whether variants in miRNAs can affect the cardiac phenotype in cardiomyopathies, such as hypertrophic cardiomyopathy (HCM), which is the most common cardiac genetic disease and cause of sudden death in young individuals.

HCM is characterized pathologically by myocyte hypertrophy, disarray, fibrosis[14] and is caused by sarcomeric protein mutations in ~60% of patients; genetic causes are unknown in ~40% of patients. Inheritance is autosomal dominant with variable penetrance and phenotypic heterogeneity. The genetic mechanisms underlying variability in penetrance and expression in HCM are not well defined [15–17]. We hypothesized that genetic variations in miRNAs that regulate cardiac hypertrophy/fibrosis might be involved in generation of the cardiac phenotype in HCM patients. We used a literature search combined with online bioinformatics tools to identify miRNAs (n = 18) that have been implicated in the pathologic features of HCM, namely, myocyte hypertrophy, cardiac fibrosis and apoptosis. Using Sanger sequence analysis of genomic DNA obtained from peripheral blood in HCM patients, we identified 11 variants in 9 of the 18 miRNAs that we investigated. We focused on the C57T single nucleotide polymorphism (SNP, rs6971711) in the miR-590 gene for two reasons. First, the C57T SNP in miR-590 is a common SNP that is only seen in African Americans; minor allele frequency of the miR-590 SNP was slightly higher in African American HCM patients when compared to ethnically-matched controls. Second, miR-590 has been reported to be an important regulator of signaling pathways involved in cardiac fibrosis/ventricular remodeling[18–20], embryonic stem cell proliferation[21], cardiac differentiation[22], metabolism [23–25], cardiac regeneration [26, 27] and atrial fibrosis/fibrillation[23]. However, it is unknown whether the miR-590 C57T SNP affects levels of miR-590-5p or miR-590-3p. In order to address this question, we used an in vitro cell culture system to investigate functional effects of this SNP. We discovered that the C57T SNP markedly reduced levels of mature miR-590-5p and miR-590-3p (when compared with the wild-type sequence) without affecting levels of pri-miR-590 and pre-miR-590. Consistent with this finding, the C57T SNP also reduced target suppression by miR-590, which suggests that presence of the miR-590 SNP could influence cell function. Since miR-590 can regulate TGF-β, Activin A and Akt signaling, SNP-induced reduction in miR-590 biogenesis could modify cardiac phenotype by de-repression of these signaling pathways.

## Materials and Methods

### Overall workflow of the studies

**Fig 1:**
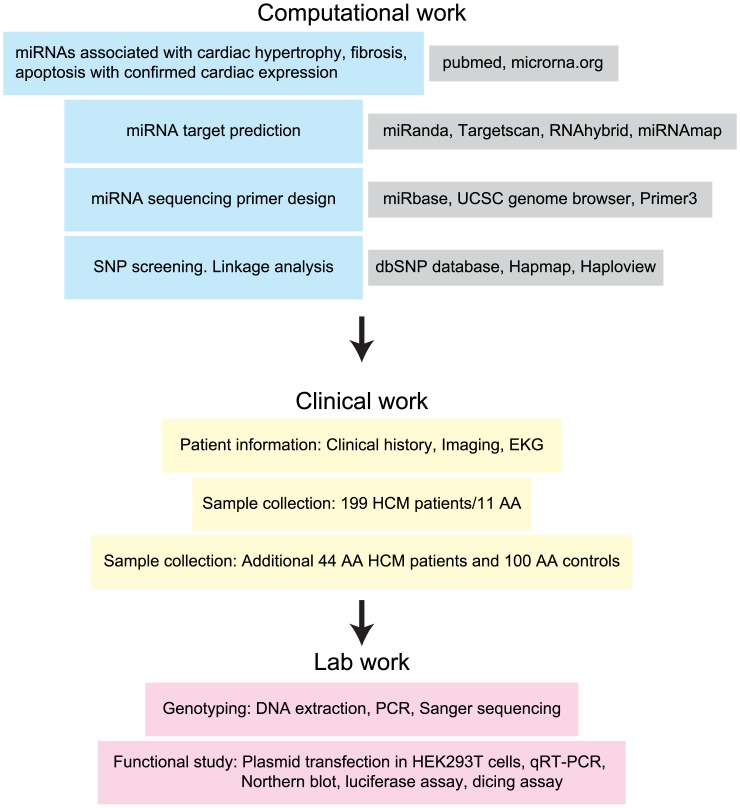
Overall study design.

The overall workflow is illustrated in Fig 1 and in the Supporting Information section. Briefly, we used literature mining to select 18 miRNAs that have been implicated in cardiac hypertrophy, fibrosis and/or apoptosis by previous experimental studies. Sanger sequencing was initially performed in 199 patients with a clinical diagnosis of HCM. Eleven of the 199 patients (5.5%) were African American and 5 of the 11 African American HCM patients carried the miR-590 C57T SNP, rs6971711 (heterozygous). Targeted genotyping for the miR-590 C57T SNP, rs6971711 was performed in a second cohort of African American HCM patients (n = 44) and controls (n = 100) to increase sample size and examine possible significance of this SNP in HCM. Functional effects of the miR-590 C57T SNP were examined using a cell culture model (human embryonic kidney cells/HEK293T).

### Identification of miRNAs for genotyping

Our goal was to identify variations in miRNAs with functional effects that could influence development of a cardiac phenotype. Since HCM patients often exhibit phenotypic variability, with varying degrees of hypertrophy, disarray and fibrosis [14], a literature search was performed using keywords that link miRNAs with the pathologic features of HCM, including cardiovascular disease, hypertrophy, fibrosis, apoptosis, metabolism, and electrophysiology. A miRNA was selected only if *1)* it was expressed in the heart; cardiac expression was confirmed using the miRNA database (http://www.microrna.org), NCBI Gene Expression Omnibus and/or published literature, and *2)* experimental studies revealed its role in generation of myocyte hypertrophy, fibrosis or apoptosis. Our literature search yielded the following 18 miRNAs which are expressed in the heart and have been previously implicated in hypertrophy, fibrosis or apoptosis in animal models or humans: miR-1-1, miR-1-2, miR-15a, miR-16-1, miR-21, miR-23a, miR-29a, miR-29b-1, miR-29b-2, miR-29c, miR-30c-1, miR-30c-2, miR-133a-1, miR-133a-2, miR-195, miR-208a, miR-208b, and miR-590 (S1 and S2 Tables)[16, 17, 23, 28–35].

Please see Supporting Information section for detailed methods.

### HCM patients

This study was approved by the Institutional Review Board at Johns Hopkins Medicine and the National Institute of Health. Written informed consent was obtained from all participants. Patients enrolled in the HCM Registry at Johns Hopkins and the NIH HCM registry were retrospectively studied if they fulfilled the standard diagnostic criteria for HCM [36], namely, left ventricular hypertrophy (septal thickness>1.5cm) in the absence of other causes such as hypertension and valvular disease. African American individuals without heart disease were used as controls. DNA was isolated from buffy coats obtained from peripheral venous blood in most subjects. HCM patients’ medical records were reviewed to obtain clinical information, including family history of HCM and results of imaging studies.

### MiRNA Sanger Sequencing

DNA analysis was performed by the Genetics Translational Technology Core at Johns Hopkins. Genomic DNA was extracted from the buffy coat of blood samples manually using the QIAamp DNA Blood Mini Kit (Qiagen), or the QiaSymphony robot. The pre-miRNA sequence and approximately 200 flanking base pairs were analyzed. The primers were designed using Primer 3 software and included M13 universal forward and reverse sequences (S5 Table). The miRNA region was amplified using HotStar Taq DNA Polymerase (Qiagen). Excess primers and dNTPs were eliminated by Exo/SAP digestion. Purified PCR products were sequenced unidirectionally using either forward or reverse M13 universal primers and the DNA sequencing kit with Big Dye Cycle Sequencing Ready Reaction Kit version 3.1 (Applied Biosystems), according to the manufacturer’s recommendations. Sequencing reaction products were separated using POP7 in a 50 cm 48 capillary array on an ABI3730 DNA Sequencer (Applied Biosystems). Sequencher 4.6 (GeneCodes) was used to align individual sequences to wild type reference sequence and identify sequence variations. All sequences were evaluated by two independent editors following criteria for identifying non-reference sequence changes validated by the Johns Hopkins DNA Diagnostic Laboratory. If a variant was detected in a single direction, then the opposite direction was sequenced for confirmation. Mfold was used to predict potential alteration of miRNA secondary structure. The Exome Aggregation Consortium (ExAC) database (http://exac.broadinstitute.org/) was used to obtain population-based allele and genotype frequency for the miR-590 C57T SNP rs6971711.

### Plasmid construction

The miR-590 gene (pri-miR-590) was cloned from human DNA using DNA primers shown in S6 Table, using the XhoI and KpnI sites, into the GV268 vector (Geneche). The C57T SNP was introduced using the QuikChangeII XL Site-Directed Mutagenesis Kit (Stratagene). To construct dual luciferase reporters, psiCheck2 (Promega) was digested with XhoI and NotI, and the dsDNA oligos listed in S6 Table were inserted.

### RNA preparation

Since endogenous expression of miR-590 is very low in HEK293T cells [37, 38], we selected this cell line for analysis of miR-590 biogenesis following exogenous introduction of plasmids. HEK293T cells (5x10^6^) were transiently transfected with 50 μg of plasmids harboring either the wild-type pri-miR-590 (pri-miR-590-WT) or C57T SNP pri-miR-590 (pri-miR-590-SNP) or empty vector (GV268) using lipofectamine 2000 (Invitrogen). Total RNA was isolated using miRVana (Life Technologies) at 72h after plasmid transfection.

### Real-time qPCR

Reverse-transcription was performed using oligo-dT primer and SuperScript II reverse transcriptase (Invitrogen). Real-time quantitative PCR was performed using Luminaris HiGreen qPCR master mix (Life Technologies) in a CFX96 system (Biorad). The sequences of the oligo primers used are listed in S6 Table.

### Northern blot

Northern blot was performed as described previously [39]: 35 μg total RNA was denatured in formamide loading buffer (98% v/v formamide, 0.1% w/v bromophenol blue, 0.1% w/v xylene cyanol, and 10mM EDTA) at 95C for 5 min and was resolved on a 0.4 mm thick, 15% denaturing polyacrylamide 7 M urea sequencing gel in 0.5×TBE (Tris-Borate-EDTA) buffer. After electrophoresis, RNA was transferred at 20 V for 1 hr to a Hybond-N+ membrane (GE healthcare) in 0.5×TBE buffer using a semi-dry transfer system (Transblot SD, Bio-Rad). The RNA was UV cross-linked (HL2000, UVP) to the membrane and pre-hybridized in Church buffer for at least 60 min at 37C. DNA oligo nucleotide probes (S6 Table) were 5′ ^32^P-radiolabeled with γ-^32^P-ATP and T4 polynucleotide kinase (NEB). After labeling, non-incorporated nucleotides were removed using a Sephadex G-25 spin column (GE healthcare). The probes were added to the Church buffer and hybridized for at least 6h at 37°C. The miR-590-3p-WT and miR-590-3p-SNP probes were hybridized at 32 and 25C, respectively, because of their lower Tm values. Membranes were washed three times for 10 min in 2×SSC containing 0.05% (w/v) SDS, subsequently exposed to Storage Phosphor Screens (GE healthcare), and analyzed using FLA-9500 (GE healthcare). Probes were stripped from the membranes in boiling 0.1% SDS solution. The membranes were re-probed with the next probe.

### Luciferase assay

S2 cell dual luciferase reporter assays using the psiCheck2 vector (Promega, Madison, WI, USA) were performed as described previously [39]. HEK293T cells (1x10^5^) were co-transfected with 20 ng of the psiCheck2 luciferase reporter plasmids and 100 ng of the pri-miR-590 plasmids (WT, SNP, empty vector), using Dharmafect Duo (GE healthcare), 24h after seeding. The media was replaced 24h after transfection. Firefly (*Photinus pyralis*) luciferase and *Renilla* luciferase activities were measured using the Dual-Glo luciferase assay system (Promega) 48h after transfection. Firefly luciferase served as the internal control.

### Dicing assay

Recombinant human Dicer was expressed and purified from Sf9 cells as described previously [39]. In vitro dicing assay was performed as previously described [39–41]. The dicing reactions contained 1 nM Dicer, 100 nM 5′ ^32^P-radiolabeled pre-miR-590 (wild-type and C57U SNP variant), 20 mM HEPES-KOH (pH 7.4), 80 mM potassium acetate, 6 mM magnesium acetate, 5 mM DTT, 0.1 mg/mL BSA, and 1 mM ATP. Aliquots of reactions were quenched by the addition of 20 volumes of formamide loading buffer, incubated at 95C for 5 min, and analyzed by electrophoresis through a denaturing polyacrylamide 7 M urea gel in 0.5xTBE buffer. Gels were dried, exposed to Storage Phosphor Screens (GE healthcare), and were analyzed with FLA-9500 (GE healthcare).

### Statistics

Continuous variables were expressed as mean± standard deviation (SD); categorical variables were presented as absolute and percentage numbers. The Student’s t-test or Mann—Whitney U test was used to test significance between groups depending on their distributions. Chi-square test was used for categorical variables. A p-value<0.05 was considered statistically significant. All statistical analyses were performed using the SPSS statistical package (v.13.0).

## Results

### HCM cohort

A total of 243 patients (mean age 46 ± 16 years; 149 men) were studied. The demographic and clinical features of the entire study population (n = 243) and the cohort of African American HCM patients (n = 55) are described in Table 1.

**Table 1:** Demographic and Clinical Characteristics of HCM patient cohort.

Patient Demographics	Total HCM cohort	Total African American HCM cohort
Number of Patients	243	55
Age (Y)	46.2± 16.8	48.5± 19.3
Male	149 (61.3%)	20 (46.5%)
BSA (m^2^)	1.87± 0.60	1.61± 0.90
*white*	166 (68.3%)	
*African American*	55 (22.6%)	
*Other*	22 (9.0%)	
**Clinical symptoms**		
CHF (NYHA class ≤II)	186 (76%)	38 (88%)
Angina	80 (32%)	22 (51%)
Syncope	34 (14%)	10 (23%)
Dyspnea	115 (47%)	21 (48%)
**Past history**		
AF	25 (10%)	8 (18%)
VT/VF	3 (1.2%)	1 (2.3%)
ICD	49 (20%)	16 (37%)
**Family history**		
Hypertrophic cardiomyopathy	114 (47%)	5 (11%)
Sudden cardiac death	35 (14%)	12 (28%)
**Echocardiography**		
LA size (cm)	3.8± 0.4	4.1± 0.7
LVOT obstruction^1^	49 (20%)	5 (11%)

BSA, body surface area; AF, atrial fibrillation; NSVT, non-sustained ventricular tachycardia; VT, sustained ventricular tachycardia; VF, ventricular fibrillation; ICD, implantable cardiac defibrillator; IVS, interventricular septal thickness; LA, left atrium; LVOT, left ventricular outflow tract

^1^LVOT obstruction is considered when left ventricular outflow tract gradient is >30 mmHg

### Identification of miRNA variants in HCM patients

Sanger sequencing of the 18 miRNA genes identified by literature search and bioinformatics analysis revealed 11 variants in 9/18 miRNAs in 89/199 HCM patients (Table 2 and Supporting Information section). Only one variant, C57T (rs6971711), was located in a mature miRNA: miR-590-3p. The allele frequency of miR-590 C57T was higher in the African American HCM cohort (8.2%, 9 of 110 alleles) compared to the control group (6.5%, 13 of 200 alleles), but the difference was not statistically significant (p = 0.5) (Table 3). Since allele frequency can vary depending on the population, we also assessed frequency of this allele in European and African populations in the Exome Aggregation Consortium (ExAC) database for comparison. Frequency of miR-590 C57T in individuals with African ancestry in the ExAC database (7.0%, 728 of 10340 alleles) was slightly lower than that seen in our African American HCM cohort (8.2%), but again the difference was not statistically significant (p = 0.5). Notably, this SNP was not seen in whites in our HCM cohort or in the European (Finnish) population in the ExAc database; minor allele frequency in the European (non-Finnish) population was very low (0.0003%, 22 of 66296 alleles) (S4 Table).

**Table 2:** Genetic variations detected in miRNA genes in the initial cohort of 199 HCM patients.

miRNA	Number of patients	miRNA gene location	SNP	Chromosome	Allele position	Distance from pre-miRNA
***Mature miRNA***						
miR-590	5	intron	rs6971711	7q11.23	g.73605599C>T	
***Pre-miRNA***						
miR-16-1	1	intron	rs72631826	13q14.2	g.50623143T>C	
***Pri-miRNA***						
miR-133a-2	82	intron	rs13040413	20q13.3	g.61162100G>A	40 bp upstream
miR-133a-2	1	intron		20q13.3	g.61162228C>T	30 bp downstream
miR-1-1	1	intron	rs6122014	20q13.3	g.61151515G>A	4 bp upstream
miR-1-2	7	intron	rs9989532	18q11.2	g.19408950A>G	26bp downstream
miR-21	1	intergenic		17q23.1	g.57918535_57918536insT +/-	98 bp upstream
miR-29b-1	1	intergenic	rs116155675	7q32.2	g.130562314A>G	25 bpupstream
miR-29b-1	1	intergenic		7q32.2	g.130562511_130562514delTCTG	222 bp upstream
miR-29b-2	1	intergenic		1q32.2	g.207975681_207975682insA +/-	113 bp downstream
miR-29c	1	intergenic		1q32.2	g.207975315G>A	46 bp upstream

Ins, insertion; del, deletion; bp, base pair

**Table 3:** Minor allele frequency in the African American HCM cohort, African American controls and individuals with African ancestry from the ExAc Database.

miRNA	SNP	Minor allele	HCM cohort	Controls	ExAc database
miR-590	rs6971711	T	0.082 (9/110)	0.065 (13/200)	0.070 (728/10340)

### MiR-590 C57T SNP rs6971711 changes G-C pair to G-U pair

**Fig 2:**
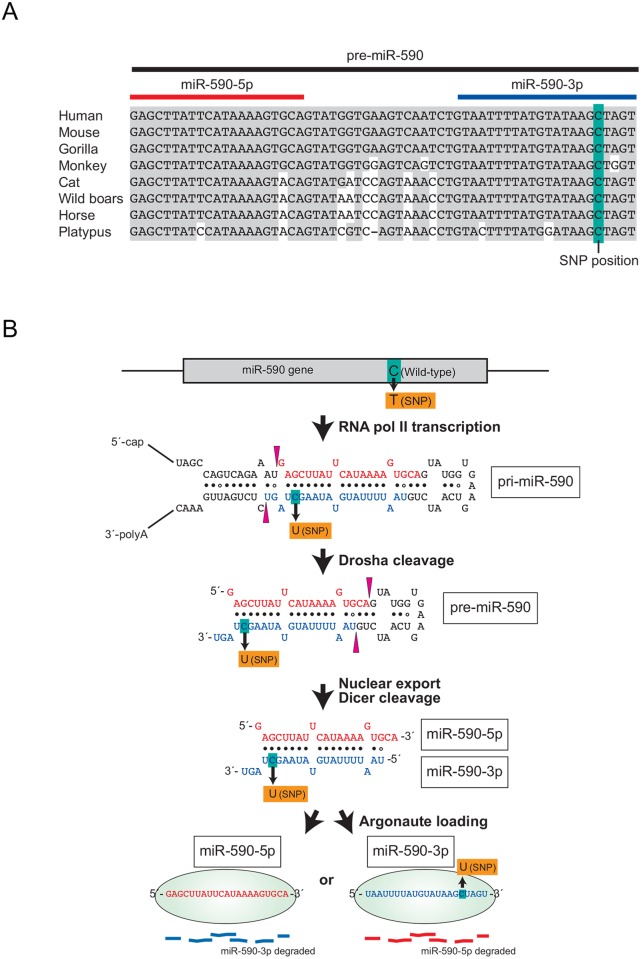
The miR-590 C57T SNP changes the conserved G3-C57 Watson-Click base pair in pri-miR-590, pre-miR-590, and miR-590 duplex to a G3-U57 wobble pair. (A) Multiple sequence alignment of pre-miR-590 from mammals. (B) miR-590 biogenesis pathway.

The miR-590 gene is located within intron 5 of the eukaryotic translation initiation factor 4H (eIF4H) gene in 7q11.23. SNP rs6971711 is located at the 57th position of pre-miR-590 (the 17th position of miR-590-3p). Therefore, it is referred to as miR-590 C57T SNP (and C57U SNP when we refer to pre-miR-590 RNA molecule). Alignment of available pre-miR-590 sequences revealed that the miR-590 C57T SNP rs6971711 is highly conserved in mammals (Fig 2A). Nucleotide C57 is located in the stem of pri-miR-590 and pre-miR-590, and forms a G-C base pair with nucleotide G3 (Fig 2B), which is also highly conserved among mammals (Fig 2A). The C57T SNP changes this conserved G-C base pair to a G-U wobble pair. This change could potentially affect miR-590 maturation, including pri-miR-590 processing by Drosha, pre-miR-590 processing by Dicer, and/or miR-590 duplex stability.

### MiR-590 C57T SNP rs6971711 reduces abundance of miR-590-5p and miR-590-3p

**Fig 3:**
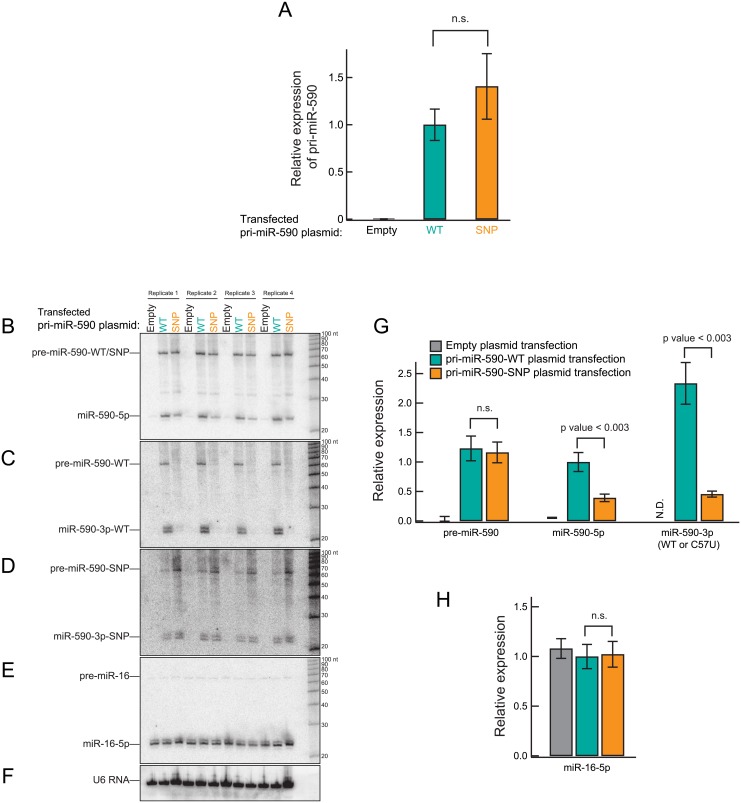
The miR-590 C57T SNP reduces levels of miR-590-5p and miR-590-3p, without affecting the levels of pri-miR-590 and pre-miR-590. (A) Quantification of pri-miR-590 by qRT-PCR normalized by GAPDH. Mean ± SD (n = 3). HEK293T cells were transfected with the pri-miR-590-WT or pri-miR-590-SNP plasmids. The empty plasmid was used as negative control. (B-F) Northern blot images for pre-miRNA, miRNA, and U6 RNA using total RNA prepared from HEK293T cells transfected with the pri-miR-590-WT or pri-miR-590-SNP plasmids. The empty plasmid was used as negative control. Four biological replicates were analyzed for each transfection plasmid. Northern probes used are perfectly complementary to miR-590-5p (A), miR-590-3p-WT (B), miR-590-3p-SNP (C), miR-16-5p (D), and U6 RNA (E). The miR-590-3p-WT probe weakly cross-hybridized to miR-590-3p-SNP, and vice versa. (G) The abundance of pre-miR-590, miR-590-5p and miR-590-3p-(WT/SNP) relative to the mean value of miR-590-5p in the WT miR-590 gene plasmid transfection conditions. (H) The abundance of miR-16-5p normalized to the mean value of the pri-miR-590-WT plasmid transfection conditions. Mean ± SD (n = 4).

First, we examined whether presence of the C57T SNP in miR-590 affects abundance of miR-590 by quantifying miR-590 production in HEK293T cells. We chose HEK293T because these cells do not express endogenous miR-590, based on high-throughput sequencing data [37, 38]. In order to examine miR-590 transcription in HEK293T cells, we quantified pri-miR-590 transcripts in total RNA obtained from HEK293T cells by qRT-PCR. We were unable to detect endogenous pri-miR-590 in HEK293T cells (Fig 3A), which permits investigation of miR-590 biogenesis by exogenously introduced plasmids. Next, we transfected HEK293T cells with plasmids containing wild-type pri-miR-590, pri-miR-590 expressing the C57T SNP or empty plasmid. We found that levels of pri-miR-590 were similar between the wild-type and the SNP pri-miR-590 plasmids transfections (p-value > 0.05), indicating that presence of the C57T SNP does not affect miR-590 transcription.

Next, we quantified the abundance of pre-miR-590, miR-590-5p and miR-590-3p by Northern blot. Using the empty plasmid as a negative control, we confirmed that almost no endogenous pre-miR-590, miR-590-5p or miR-590-3p are detected in HEK293T cells (Fig 3B and 3C). We quantified the pre-miR-590 (WT, SNP) and miR-590-5p levels using a miR-590-5p probe (Fig 3B and 3G), normalized by the internal loading control, U6 RNA (Fig 3F). The C57T SNP did not significantly affect levels of pre-miR-590 (p-value > 0.05) (Fig 3B and 3G). In contrast, the C57T SNP markedly reduced (p-value < 0.003) levels of mature miR-590-5p. The amount of miR-590-5p produced from the pri-miR-590-SNP plasmid was 39 ± 16% of that produced from the pri-miR-590-WT plasmid.

Unlike miR-590-5p, the relative abundance of miR-590-3p-WT and miR-590-3p-SNP cannot be quantified directly using a probe, because the SNP affects probe hybridization. To overcome this issue, we quantified the ratio of miR-590-5p to pre-miR-590 in each sample (Fig 3B). Then we measured the ratio of miR-590-3p-WT to pre-miR-590-WT following transfection with the pri-miR-590-WT plasmid, using the miR-590-3p-WT probe (Fig 3C). Similarly, we measured the ratio of miR-590-3p-SNP to pre-miR-590-SNP following transfection with the pri-miR-590-SNP plasmid, using the miR-590-3p-SNP probe (Fig 3D). Using these three ratios and the pre-miR-590 levels normalized by U6 RNA determined in Fig 3B, we calculated the relative abundance of miR-590-3p-WT and miR-590-3p-SNP (Fig 3G). The C57T SNP significantly (p-value < 0.003) reduced levels of miR-590-3p: miR-590-3p produced from the pri-miR-590-SNP plasmid was 20 ± 2% of that produced from the pri-miR-590-WT plasmid. Levels of endogenous miR-15-5p, miR-16-5p, miR-17-5p, and miR-25-3p were examined as controls. We did not observe differences in levels of these miRNAs following transfection with plasmids expressing miR-590 WT and SNP (p-value > 0.05) (Fig 3E and 3G and S1 Fig). We concluded that the miR-590 C57T SNP reduces levels of both miR-590-5p and miR-590-3p, without affecting levels of pri-miR-590 and pre-miR-590.

### MiR-590 C57T SNP rs6971711 does not affect pre-miR-590 processing by recombinant Dicer in the test tube

**Fig 4:**
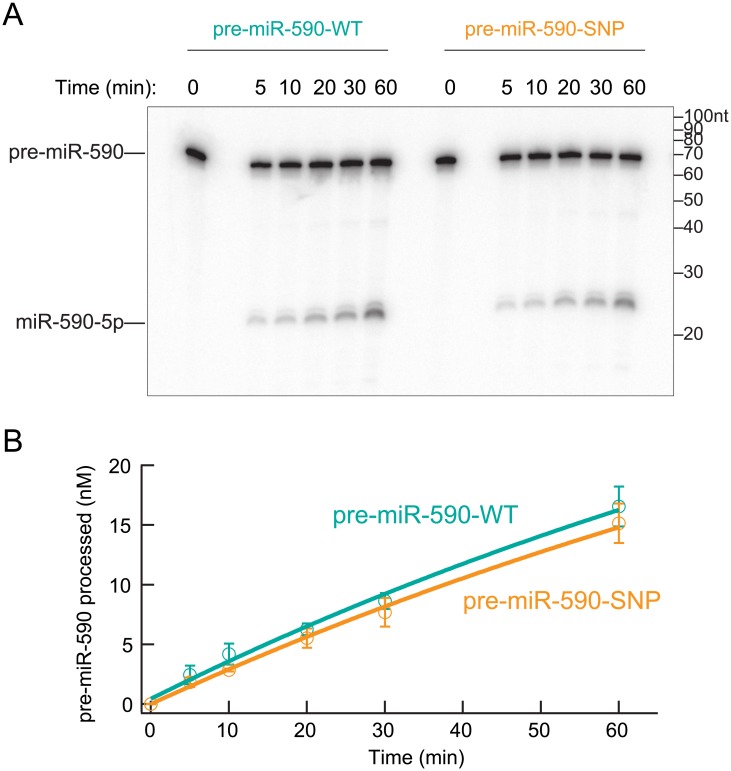
The miR-590 C57T SNP does not affect pre-miR-590 processing by recombinant Dicer in test tube. (A) Representative image of pre-miR-590 (100 nM) processing assay by recombinant human Dicer (1 nM) in test tube. (B) Quantification of three independent replicates of the experiment in (A). Mean ± SD (n = 3).

Since the miR-590 C57T SNP reduced levels of miR-590-5p and miR-590-3p, without affecting levels of pri-miR-590 and pre-miR-590 (Fig 3), we assessed whether this SNP negatively affects pre-miR-590 processing by Dicer. We tested this possibility in vitro using recombinant human Dicer, 5′ ^32^P radiolabeled WT pre-miR-590 and pre-miR-590 containing the SNP (C57U). We found that Dicer processed the C57U SNP-containing pre-miR-590 as efficiently as the WT type pre-miR-590 (Fig 4), which led us to conclude that this SNP does not affect processing of pre-miR-590 by recombinant Dicer in the test tube.

### MiR-590 C57T SNP rs6971711 reduces target suppression by miR-590-5p

**Fig 5:**
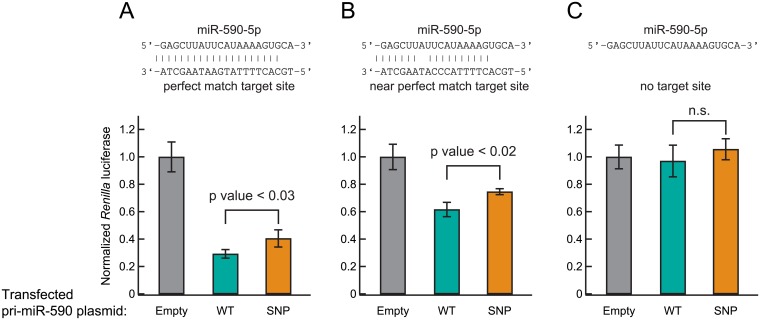
The miR-590 C57T SNP reduces target suppression by miR-590. Silencing of *Renilla* luciferase reporters bearing 3′ UTR target sites for miR-590-5p. Reporters containing four tandemmiR-590-5p perfect match sites with t1A (A), four tandemmiR-590-5p near perfect match sites with mismatches at positions 9–11 and t1A (B), and no miR-590 target site (C). HEK293T cells were cotransfected with the luciferase reporter plasmids and the pri-miR-590-WT or pri-miR-590-SNP plasmid. The empty plasmid was used as a negative control. *Renilla* luciferase expression relative to the firefly luciferase internal control is shown. Mean ± SD (n = 4).

Since the C57T SNP reduced levels of miR-590-5p and miR-590-3p, we expected that the SNP would reduce target suppression by miR-590. We tested this using the dual luciferase reporter assay in HEK293T cells. We constructed *Renilla reniformis* (*Rr*) luciferase reporters which have in their 3′ UTR, (1) four tandem perfect match target sites of miR-590-5p (Fig 5A), (2) four tandem near perfect match target sites of miR-590-5p, in which positions 9–11 are mismatched (Fig 5B), or (3) no miR-590-5p target sites (Fig 5C). Co-transfection of the perfect match target sites reporter plasmid with the pri-miR-590-WT plasmid suppressed *Rr* firefly expression to 29 ± 3% of the empty vector control (Fig 5A). In contrast, the pri-miR-590-SNP plasmid suppressed *Rr* luciferase expression to 40 ± 6% of the empty vector control. The difference between the WT and SNP was significant (p-value < 0.03). Similarly, co-transfection of near perfect match target sites reporter plasmid with the pri-miR-590-WT plasmid suppressed *Rr* luciferase expression to 62 ± 5% of the empty vector control, while the pri-miR-590-SNP plasmid co-transfection suppressed *Rr* luciferase expression to 75 ± 2% of the empty vector (Fig 5B). The difference between the WT and SNP was significant (p-value < 0.02). The control, no target site reporter was not suppressed by pri-miR-590-WT or pri-miR-590-SNP plasmids (Fig 5C). We concluded that the miR-590 C57T SNP reduces miR-590-5p target suppression, which is consistent with our results that this SNP reduces abundance of miR-590-5p (Fig 3).

### MiR-590 is expressed in human cardiac myocytes

**Fig 6:**
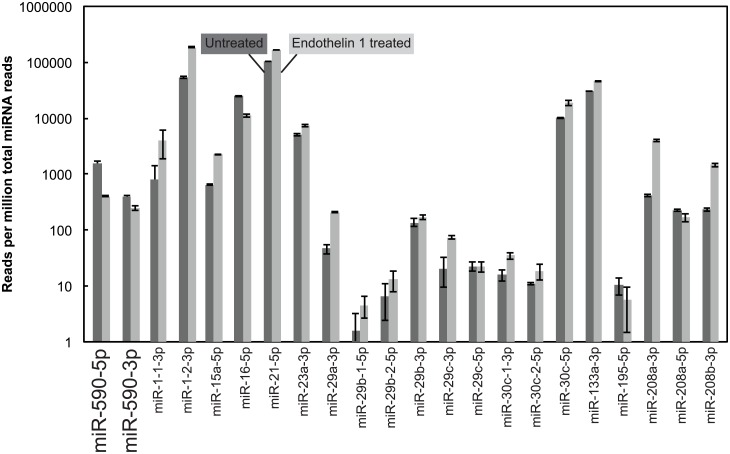
Expression of selected miRNAs in human cardiac myocytes derived from induced pluripotent stem cells (IPSC-CMs). The high-throughput miRNA-sequencing data (GSE60292) in Aggarwal et al [44] were analyzed. The dark gray bars and light gray bars represent IPSC-CMs with and without Endothelin-1 treatment, respectively. Mean ± SD (n = 3). Endothelin-1 (ET-1) treatment is commonly used to stimulate cardiac myocyte hypertrophy [44, 49, 59].

Using a literature search, we learned that miR-590-5p and miR-590-3p are expressed in human hearts (GEO datasets GSE53080 [42], GSE46224 [43], and GSE36946) and cardiac myocytes derived from human induced pluripotent stem cells (IPSC-CMs) [44]. We analyzed miRNA-sequencing data (GSE60292 [44]) from human IPSC-CMs with/without endothelin-1 treatment (which induces cardiac hypertrophy). We observed that majority of the 18 miRNAs that we selected for genotyping are expressed in human IPSC-CMs. Interestingly, induction of hypertrophy by endothelin-1 treatment significantly reduced levels of miR-590-5p and miR-590-3p in IPSC-CMs (p-value < 0.003 and < 0.002, respectively) (Fig 6), suggesting a possible role for miR-590 in cardiac hypertrophy.

## Discussion

MiRNAs are important regulators of multiple genes via post-transcriptional gene silencing and of physiologic as well as pathologic processes such as cardiac development, hypertrophy, fibrosis, proliferation, apoptosis, and cancer. Previous studies have revealed that occurrence of SNPs or variants in human pre-miRNA sequences is relatively rare: ~10% of human pre-miRNAs have documented SNPs and <1% of human miRNAs have documented SNPs in the seed region, which is crucial for target gene regulation [45]. Base-pairing between the highly conserved, 5′ proximal seed region (residues 2–8) of miRNA and the 3' UTR of target mRNA is important for miRNA:mRNA binding and silencing [4, 5]. Additionally, sequences outside the miRNA seed region can also impact target silencing efficiency and/or the spectra of targeted transcripts[6–8]. Our study revealed a SNP in the 17th position of mature miR-590-3p. Experimental studies indicated a ‘*loss of function effect’* of the miR-590 C57T SNP (rs6971711) on mature miR-590 abundance, which is most likely due to SNP-induced change in the G-C Watson-Click base pair within the pri- and pre-miR-590 stem and miR-590 duplex into a G-U wobble base pair. It is possible that this change alters geometry of the RNA helix and thus affects recognition of the pri- and/or pre-miR-590 stem and miR-590 duplex by enzymes or RNA-binding proteins [46–48], while the stems of most of pri-miRNAs and pre-miRNAs, including pri-miR-590 and pre-miR-590 (Fig 2B) contain G-U wobble base pairs, mismatched base pairs, and/or bulged nucleotides. Where in the miR-590 biogenesis step, does the C57T SNP exert its effect? Considering that only mature miR-590-5p and -3p are reduced, but pri-miR-590 and pre-miR-590 levels are unaffected, it appears that the SNP negatively affects miR-590 maturation downstream of pre-miR-590 production by Drosha in nucleus. The possibilities include the following: C57T SNP may reduce (i) transport of pre-miR-590 from nucleus to cytoplasm by Exportin-5, (ii) processing of pre-miR-590 into miR-590 duplex by Dicer, (iii) loading of miR-590 duplex to Argonaute and/or (iv) stability of the miR-590 duplex. We performed an in vitro pre-miR-590 processing assay using recombinant Dicer protein to test possibility (ii) and observed no effect of the SNP on pre-miR-590 processing. Future work is needed to test if this is the case in cells and in vivo. We also tested possibility (iv) using an in vitro system, and found that the miR-590 C57T SNP did not affect stability of the miR-590 duplex in HEK293T cell lysate; the WT and SNP miR-590 duplex were degraded at similar rates by cellular RNases present in the HEK293T cell lysate (S2 Fig). Further studies are required to identify the specific step(s) in the miR-590 biogenesis pathway that is negatively affected by the C57T SNP.

MiR-590-5p and miR-590-3p are expressed in human IPSC-CMs and in the human heart (Fig 6) [42–44]. MiR-590 regulates signaling pathways (TGF-β, activin, Akt) which are involved in cardiac fibrosis/remodeling [18–20], embryonic stem cell proliferation/cardiac differentiation[22] and metabolism by suppressing TGF-β receptor II (TGFβRII)[23], Activin receptor 2a (Acvr2a) and PTEN (phosphatase and tensin homolog) [24, 25] expression, respectively. Interestingly, overexpression of miR-590-3p stimulated neonatal myocyte proliferation and cardiac regeneration following myocardial infarction [26, 27], whereas miR-590 down-regulation was associated with atrial fibrosis and atrial fibrillation [23]. Notably, endothelin-1 treatment which induces cardiac hypertrophy, reduced levels of miR-590-5p and miR-590-3p in human IPSC-CMs (Fig 6) [44, 49]. Taken together, this data suggests a role for miR-590 in cardiac physiology and disease.

Mutations or SNPs in miRNAs can cause disease via two main mechanisms: First, variations in miRNA coding regions, especially the seed region, can act as causal mutations in inherited disease. For example, a mutation in the seed region of miR-96 was segregated with human hearing loss in a large family and this was further reproduced using animal models [10]. MiRNAs may also serve as modifier genes[50]. SNPs (or variants) in miRNA genes have been demonstrated to influence miRNA expression, processing and/or maturation thereby affecting downstream gene targeting [6]. A recent study demonstrated that miR-499 is up-regulated in cardiac hypertrophy and cardiomyopathy [51] and a variant located outside the seed region (miR-499-5p c*17*) conveyed a favorable impact on the cardiac phenotype when compared to wild-type miR-499-5p by altering the target gene profile [8]. Interestingly, the location of the SNP rs6971711 in mature miR-590-3p (detected in our study) and the miR-499-5p variant are identical; both SNPs are located at the 17th nucleotide position of the respective mature miRNA. Since the miR-590 C57T SNP was observed in 6.5% of controls, it is unlikely to be a causal gene in cardiomyopathies. But it could be a modifier of the cardiac phenotype in heart disease including HCM. SNP-induced reduction of miR-590 levels could lead to de-repression of TGFβRII (target of miR-590-5p[23]) and Acvr2a (target of miR-590-5p/3p), receptors involved in TGF-β and Activin A signaling, respectively, which in turn could influence cardiac hypertrophy and fibrosis (cardiac remodeling) and thus, clinical outcomes in the setting of cardiomyopathies and following myocardial infarction [18].

### Clinical implications

African-Americans with heart disease have higher cardiovascular mortality rates [52], disproportionately higher rates of heart failure [53–55] and higher mortality [56] in the setting of heart failure, when compared to whites. However, the mechanisms underlying differences in outcomes between African Americans and whites is unknown. Since the C57T SNP in miR-590 is common in African-Americans and can regulate TGF-β signaling, an important regulator of cardiac fibrosis/ventricular remodeling [57, 58], presence of the miR-590 C57T SNP could promote cardiac fibrosis by de-repression of TGF-β signaling.

### Limitations

The small number of African American HCM patients in our study precluded assessment of the relationship between the common miR-590 C57T SNP and cardiac phenotype. This result is not unexpected because common genetic polymorphisms are known to have small effects on disease phenotype. Future basic and epidemiologic studies in large numbers of African Americans are needed to investigate effect of the miR-590 C57T SNP on myocyte physiology and cardiac fibrosis/function.

## Supporting Information

S1 Fig The pri-miR-590 (WT/SNP) plasmid transfection does not affect levels of endogenous miRNAs. (A) Northern blot images for endogenous miR-15-5p, miR-17-5p, and miR-25-3p, using total RNA prepared from HEK293T cells transfected with the pri-miR-590-WT or pri-miR-590-SNP plasmids. The empty plasmid was used as a negative control. Four biological replicates were analyzed for each plasmid transfection condition. (B) The abundance of miR-15-5p, miR-17-5p, and miR-25-3p normalized to mean value of the wild-type pri-miR-590 plasmid transfection condition. Mean ± SD (n = 4).
(EPS)

S2 Fig The miR-590 C57T SNP does not affect stability of miR-590 duplex in vitro. (A) Representative image of in vitro miRNA degradation assay by cellular RNases present in HEK293T cell lysate. 10 or 100 nM of RNA samples containing miR-590-5p and miR-590-3p-(WT/SNP) were incubated in HEK293T cell lysate. The red star indicates the 5′ ^32^P-radiolabeled strand. (B, C) Quantification of three independent replicates of the experiment in (A). (B) 10 nM miRNA samples. (C) 100 nM miRNA samples. Mean ± SD (n = 3). The colors correspond to those of the rectangles surrounding the miR-590 duplexes in (A).
(EPS)

S1 File Supplementary Methods and Results. (DOCX)

S1 Table Selected miRNAs and their expression in heart disease. (DOCX)

S2 Table MiRNA target prediction results by three algorithms. (DOCX)

S3 Table Distance between selected GWAS cardiac phenotypic markers and variants found in HCM population. (DOCX)

S4 Table Comparison of population-based allele frequency between the HCM cohort and ExAc Database. (DOCX)

S5 Table Primers for miRNA sequencing. (DOCX)

S6 Table DNA and RNA oligo sequences for functional studies. (DOCX)
