# Supplementary material for: Common miR-590 Variant rs6971711 Present Only in African Americans Reduces miR-590 Biogenesis
Source: PLoS One. 2016 May 19;11(5):e0156065. doi: 10.1371/journal.pone.0156065 (PMC4873136; doi:10.1371/journal.pone.0156065)
Supplement: S1 File — (DOCX) [file pone.0156065.s003.docx]

**Supplementary Methods and Results**

# Methods

Identification of miRNAs and bioinformatics studies

**Rationale for use of bioinformatics**: Recent studies of miRNA profiling in HCM patients and transgenic mouse models have identified patterns that are unique to each HCM model/mutation [[1-3](#_ENREF_1)]. However, the role of genetic variations in miRNAs has not been extensively investigated. We performed a literature search to identify candidate miRNAs that may play a role in the development of the HCM phenotype. We used this approach for the following reasons: 1) obtaining left ventricular tissue from patients, for miRNA profiling, prior to development of the HCM phenotype is fraught with ethical issues; 2) results obtained from myectomy samples in symptomatic patients with advanced disease may not be reflective of the pre-hypertrophic stage. In fact, miRNA profiling in a mouse model of HCM confirmed changes in miRNA expression profiles during different stages of the development of cardiac hypertrophy [[1](#_ENREF_1)]; 3) While several transgenic mouse models expressing mutations seen in HCM patients have been created, their miRNA expression profiles have not been compared. Our goal was to identify variations in miRNAs with functional effects that could influence development of the cardiac phenotype.

**Methods**: Since HCM patients often exhibit phenotypic variability, with varying degrees of hypertrophy, disarray and fibrosis [[4](#_ENREF_4)], a literature search was performed using keywords that link miRNAs with the pathologic features of HCM including cardiovascular disease, hypertrophy, fibrosis, apoptosis, metabolism, and electrophysiology. MiRNAs were selected only if 1) they were expressed in the heart; cardiac expression was confirmed in the miRNA database (http://www.microrna.org), NCBI Gene Expression Omnibus, and/or published literatures and 2) experimental studies implicated their role in the pathophysiology of myocyte hypertrophy, fibrosis or apoptosis. Our literature search yielded the following miRNAs which are expressed in the heart and have been previously implicated in hypertrophy, fibrosis or apoptosis in animal models or humans: miR-1-1, miR-1-2, miR-15a, miR-16-1, miR-21, miR-23a, miR-29a, miR-29b-1, miR-29b-2, miR-29c, miR-30c-1, miR-30c-2, miR-133a-1, miR-133a-2, miR-195, miR-208a, miR-208b, and miR-590 (S1 Table) [[5-15](#_ENREF_5)].

In order to establish a connection between identified miRNAs and target genes that could lead to the HCM phenotype and arrhythmias, the 3′ untranslated regions (UTRs) from the human genome were scanned for potential target sites, with an emphasis on genes affecting fibrosis, hypertrophy, apoptosis, cardiac ion channels, cardiac conduction and metabolism. To reduce the false positive rate and increase reliability, three different algorithms were used, namely Targetscan (http://www.targetscan.org/), which emphasizes perfect base-pairing in the seed region and sequence conservation, RNAhybrid (http://bibiserv.techfak.uni-bielefeld.de/rnahybrid/), which is focused on finding the minimum free energy hybridization of miRNA-mRNA, and miRanda (http://www.miranda-im.org/), which uses sequence complementarity and free energy of miRNA-mRNA duplexes.MiRNAs and selected target genes identified by all three prediction algorithms are summarized in S2 Table.

**Phenotypic association analysis using GWAS database**

We used (http://www.genome.gov/gwastudies/) to select cardiac phenotypes and associated SNPs (from GWAS studies) that were in the same chromosomal region as SNPs identified in HCM patients. The following keywords were used in the search: hypertrophy, atrial fibrillation, electrical conduction measurements, electrocardiogram traits, hypertension, metabolic traits, resting heart rate, sudden cardiac arrest, atrial ventricular conduction, coronary artery disease, treadmill test, heart failure, left ventricular mass, myocardial infarction early onset, PR interval, QT interval, ventricular conduction, hypertension, systolic blood pressure, diastolic blood pressure, cardiovascular disease risk factors and blood pressure. The SNAP program (<http://www.broadinstitute.org/mpg/snap/ldsearch.php>) was used to assess if the SNPs identified in this HCM cohort were in LD with SNPs previously linked to cardiac disease using GWAS. Identified GWAS SNPs were used to search for LD SNPs. For SNAP parameters, all four SNP datasets were checked, r^2^ threshold was set to 0.8, distance limit was 500 kb and matched populations were used.

**In vitro miR-590 degradation assay**

HEK293T cell lysate was prepared by homogenizing the cells in lysis buffer containing 30 mM HEPES-KOH (pH 7.4), 100 mM potassium acetate, 2 mM magnesium acetate, 2 mM DTT, 1 mM ATP, and protease inhibitors using glass homogenizer. The lysate was centrifuged at 21,000 g for 10 min at 4C and supernatant was recovered. The cell lysate was flash frozen in liquid nitrogen and stored in -80C until used. First, 10 nM of 5′ ^32^P-radiolabeled miR-590 strand was mixed with 12 nM of the cold opposite strand of miR-590, heated at 95C for 1min and cooled down to room temperature. The miR-590 mix was incubated in the HEK293T cell lysate at 37C. Aliquots of reactions were quenched by the addition of 20 volumes of formamide loading buffer, incubated at 95C for 5 min and analyzed by electrophoresis through a denaturing polyacrylamide 7 M urea gel in 0.5xTBE buffer. Gels were dried, exposed to storage phosphorscreens (GE healthcare), and were analyzed with FLA-9500 (GE healthcare).

# Results

Identification of variants in HCM patients

We identified 11 variants in 9 miRNAs in 89/199 HCM patients in the initial cohort; 8 were SNPs and 3 were insertions or deletions (Table 2). Of the SNPs, 6 were located in the primary miRNA (pri-miRNA) region, 1 was in the pre-miRNA region, and 1 was in the mature miRNA region, but not in the seed region. Three of the 6 SNPs located in the pri-miRNA region (rs6122014, rs13040413, rs9989532, rs116155675) and the 2 SNPs in the pre- and mature miRNA region (rs72631826, rs6971711) were registered in dbSNP (Table 2). Eight patients had ≥2SNPs detected (three patients with miR133a-2 and miR-590; two patients with miR-1-2 and miR-133a-2; one patient with miR-16-1 and miR-590; one patient with miR-1-1, miR-1-2, and miR-133a-2; one patient with miR-1-2 and miR-29c, miR-133a-2, and miR-590) - 2 were whites, 5 were African Americans.

A search of the GWAS database using select cardiac phenotypic markers revealed 15 SNPs in 11 phenotypes that were in the same chromosome region as our miRNA-SNPs. However, none of our miRNA-SNPs was within the 500 kb range of SNPs detected by GWAS, suggesting a lack of significant LD (S3 Table).

Since allele frequency differences between populations could contribute to phenotypic differences, we focused on the allele frequency in whites and African-Americans who mainly composed our study population. The corresponding populations in the ExAc database that we used for comparison were African and European (Finish and non-Finish). Comparison of the minor allele frequency between our HCM cohort and corresponding populations in the ExAc database were shown in S4 Table.

# References

1. Bagnall RD, Tsoutsman T, Shephard RE, Ritchie W, Semsarian C (2012) Global microRNA profiling of the mouse ventricles during development of severe hypertrophic cardiomyopathy and heart failure. PLoS One 7: e44744.

2. Kuster DW, Mulders J, Ten Cate FJ, Michels M, Dos Remedios CG, et al. (2013) MicroRNA transcriptome profiling in cardiac tissue of hypertrophic cardiomyopathy patients with MYBPC3 mutations. J Mol Cell Cardiol 65: 59-66.

3. Leptidis S, El Azzouzi H, Lok SI, de Weger R, Olieslagers S, et al. (2013) A deep sequencing approach to uncover the miRNOME in the human heart. PLoS One 8: e57800.

4. Elliott P, McKenna WJ (2004) Hypertrophic cardiomyopathy. Lancet 363: 1881-1891.

5. Care A, Catalucci D, Felicetti F, Bonci D, Addario A, et al. (2007) MicroRNA-133 controls cardiac hypertrophy. Nat Med 13: 613-618.

6. van Rooij E, Sutherland LB, Qi X, Richardson JA, Hill J, et al. (2007) Control of stress-dependent cardiac growth and gene expression by a microRNA. Science 316: 575-579.

7. van Rooij E, Sutherland LB, Liu N, Williams AH, McAnally J, et al. (2006) A signature pattern of stress-responsive microRNAs that can evoke cardiac hypertrophy and heart failure. Proc Natl Acad Sci U S A 103: 18255-18260.

8. Yang B, Lin H, Xiao J, Lu Y, Luo X, et al. (2007) The muscle-specific microRNA miR-1 regulates cardiac arrhythmogenic potential by targeting GJA1 and KCNJ2. Nat Med 13: 486-491.

9. Zhao Y, Ransom JF, Li A, Vedantham V, von Drehle M, et al. (2007) Dysregulation of cardiogenesis, cardiac conduction, and cell cycle in mice lacking miRNA-1-2. Cell 129: 303-317.

10. van Rooij E, Sutherland LB, Thatcher JE, DiMaio JM, Naseem RH, et al. (2008) Dysregulation of microRNAs after myocardial infarction reveals a role of miR-29 in cardiac fibrosis. Proc Natl Acad Sci U S A 105: 13027-13032.

11. Callis TE, Pandya K, Seok HY, Tang RH, Tatsuguchi M, et al. (2009) MicroRNA-208a is a regulator of cardiac hypertrophy and conduction in mice. J Clin Invest 119: 2772-2786.

12. Duisters RF, Tijsen AJ, Schroen B, Leenders JJ, Lentink V, et al. (2009) miR-133 and miR-30 regulate connective tissue growth factor: implications for a role of microRNAs in myocardial matrix remodeling. Circ Res 104: 170-178, 176p following 178.

13. Shan H, Zhang Y, Lu Y, Zhang Y, Pan Z, et al. (2009) Downregulation of miR-133 and miR-590 contributes to nicotine-induced atrial remodelling in canines. Cardiovasc Res 83: 465-472.

14. Terentyev D, Belevych AE, Terentyeva R, Martin MM, Malana GE, et al. (2009) miR-1 overexpression enhances Ca(2+) release and promotes cardiac arrhythmogenesis by targeting PP2A regulatory subunit B56alpha and causing CaMKII-dependent hyperphosphorylation of RyR2. Circ Res 104: 514-521.

15. Matkovich SJ, Wang W, Tu Y, Eschenbacher WH, Dorn LE, et al. (2010) MicroRNA-133a protects against myocardial fibrosis and modulates electrical repolarization without affecting hypertrophy in pressure-overloaded adult hearts. Circ Res 106: 166-175.
