# Supplementary material for: Common miR-590 Variant rs6971711 Present Only in African Americans Reduces miR-590 Biogenesis
Source: PLoS One. 2016 May 19;11(5):e0156065. doi: 10.1371/journal.pone.0156065 (PMC4873136; doi:10.1371/journal.pone.0156065)
Supplement: S1 Table — (DOCX) [file pone.0156065.s004.docx]

**Supplementary Table 1. Selected miRNAs and their expression in heart disease**

| **miRNAs** | **Cardiac phenotype** | **Expression change** | **Animal model** | **Human model** | **Further comfirmed*** |
| --- | --- | --- | --- | --- | --- |
| miR-1-1 | hypertrophy, arrhythmia,apoptosis, proliferation | up and down | TAB | DCM,HF | 1 |
| miR-1-2 | hypertrophy, arrhythmia,apoptosis,  proliferation | up and down | TAB | DCM,HF | 1 |
| miR-133a-1 | hypertrophy,arrhythmia, fibrosis,proliferation | down | TAB | DCM,ICM,HF | 1 |
| miR-133a-2 | hypertrophy, arrhythmia, fibrosis,proliferation | down | TAB | DCM,ICM,HF | 1 |
| miR-30C-1 | fibrosis | down | Ischemia  TAB | DCM,HF | 2 |
| miR-30C-2 | fibrosis | down | Ischemia  TAB | DCM,HF | 2 |
| miR-29a | fibrosis | down | Ischemia  TAB | NIDCM,HF | 1 |
| miR-29b-1 | fibrosis | down | Ischemia  TAB | NIDCM,HF | 1 |
| miR-29b-2 | fibrosis | down | Ischemia  TAB | NIDCM,HF | 1 |
| miR-29c | fibrosis | down | Ischemia  TAB | NIDCM,HF | 1 |
| miR-23a | hypertrophy | up | TAB | DCM,ICM,HF | 1 |
| miR-21 | hypertrophy, apoptosis, fibrosis | up | Ischemia  TAB | HF | 1 |
| miR-208a | hypertrophy, arrhythmia | up |  | DCM | 1 |
| miR-208b | hypertrophy | up |  | DCM | 1 |
| miR-195 | hypertrophy | up | Ischemia  TAB | DCM,ICM,HF | 1 |
| miR-590 | fibrosis, myocyte proliferation | Up and down | Myocardial infarction | HCM | 2 |
| miR-15a | BCL2, apoptosis (only leukemia), cardiac metabolism | down |  |  | 2 |
| miR-16-1 | BCL2, apoptosis (only leukemia) | down |  |  | 2 |

TAB, thoracic Aortic Banding; DCM, dialated cardiomyopathy; ICM, ischemic cardiomyopathy; NIDCM, non ischemic dialated cardiomyopathy; HF, heart failure; af, atrial fibrillation; * the cardiac function of miRNAs were further confirmed by in vitro (1) or in vivo (2) experiment
