# Supplementary material for: Common miR-590 Variant rs6971711 Present Only in African Americans Reduces miR-590 Biogenesis
Source: PLoS One. 2016 May 19;11(5):e0156065. doi: 10.1371/journal.pone.0156065 (PMC4873136; doi:10.1371/journal.pone.0156065)
Supplement: S2 Table — (DOCX) [file pone.0156065.s005.docx]

**Supplementary Table 2**. MiRNA target prediction results by 3 algorithms

| Function | Description | Symbol | miRNA |
| --- | --- | --- | --- |
| Electrophysiology | Gap junction alpha-1 protein (Connexin-43) (Cx43) | [GJA1](http://www.ensembl.org/Homo_sapiens/searchview?species=;idx=;q=GJA1) | 1* |
|  | Calcium channel, voltage-dependent, alpha 2/delta subunit 2 isoform b | [CACNA2](http://www.ensembl.org/Homo_sapiens/searchview?species=;idx=;q=CACNA2D2) | 133a |
|  | Potassium channel subfamily T member 1 (KCa4.1). | [KCNT1](http://www.ensembl.org/Homo_sapiens/searchview?species=;idx=;q=KCNT1) | 195 |
|  | Potassium voltage-gated channel subfamily C member 4 | [KCNC4](http://www.ensembl.org/Homo_sapiens/searchview?species=;idx=;q=KCNC4) | 195 |
|  | Voltage-dependent N-type calcium channel subunit alpha-1B | [CACNA1B](http://www.ensembl.org/Homo_sapiens/searchview?species=;idx=;q=CACNA1B) | 29c |
|  | Gap junction alpha-1 protein | [GJA1](http://www.ensembl.org/Homo_sapiens/searchview?species=;idx=;q=GJA1) | 30 |
|  | Kv channel-interacting protein 4 (KChIP4) | [KCNIP4](http://www.ensembl.org/Homo_sapiens/searchview?species=;idx=;q=KCNIP4) | 30 |
|  | Sodium channel protein type 7 subunit alpha (Sodium channel protein type VII subunit alpha) | [SCN7A](http://www.ensembl.org/Homo_sapiens/searchview?species=;idx=;q=SCN7A) | 155 |
|  | Potassium channel subfamily T member 1 | [KCNT1](http://www.ensembl.org/Homo_sapiens/searchview?species=;idx=;q=KCNT1) | 16-1 |
| Metabolism | ATP binding domain 1 family, member C | [ABCB6](http://www.ensembl.org/Homo_sapiens/searchview?species=;idx=;q=ABCB6) | 195 |
|  | ATP-binding cassette sub-family F member 2 | [ATP5G1](http://www.ensembl.org/Homo_sapiens/searchview?species=;idx=;q=ATP5G1) | 195 |
|  | Mitochondrial ATP-binding cassette sub-family B member 6 | [ATPBD1C](http://www.ensembl.org/Homo_sapiens/searchview?species=;idx=;q=ATPBD1C) | 29c |
|  | ATP synthase lipid-binding protein, mitochondrial precursor | [ABCF2](http://www.ensembl.org/Homo_sapiens/searchview?species=;idx=;q=ABCF2) | 29c |
|  | ATP-binding cassette sub-family A member 12 | [ABCA12](http://www.ensembl.org/Homo_sapiens/searchview?species=;idx=;q=ABCA12) | 30 |
|  | ATP binding domain 1 family, member C | [ATPBD1C](http://www.ensembl.org/Homo_sapiens/searchview?species=;idx=;q=ATPBD1C) | 15a |
| Contractile proteins | E3 ubiquitin-protein ligase MYLIP | [MYLIP](http://www.ensembl.org/Homo_sapiens/searchview?species=;idx=;q=MYLIP) | 133a |
|  | Myosin heavy chain 11 | [MYH11](http://www.ensembl.org/Homo_sapiens/searchview?species=;idx=;q=MYH11) | 195 |
|  | Myosin light chain kinase, smooth muscle | [MYLK](http://www.ensembl.org/Homo_sapiens/searchview?species=;idx=;q=MYLK) | 195 |
|  | Tropomyosin alpha-3 chain | [TPM3](http://www.ensembl.org/Homo_sapiens/searchview?species=;idx=;q=TPM3) | 195 |
|  | Tropomyosin-1 alpha chain | [TPM1](http://www.ensembl.org/Homo_sapiens/searchview?species=;idx=;q=TPM1) | 29c |
|  | Myosin light chain kinase, smooth muscle | [MYLK](http://www.ensembl.org/Homo_sapiens/searchview?species=;idx=;q=MYLK) | 155 |
|  | Tropomyosin-3 alpha chain | [TPM3](http://www.ncbi.nlm.nih.gov/gene/7170) | 16-1 |
| Fibrosis | Fibronectin precursor (FN) | [FN1](http://www.ensembl.org/Homo_sapiens/searchview?species=;idx=;q=FN1) | 1* |
|  | Collagen alpha-2(IX) chain precursor. | [COL9A2](http://www.ensembl.org/Homo_sapiens/searchview?species=;idx=;q=COL9A2) | 195 |
|  | Collagen alpha-1(XVI) chain precursor. | [COL16A1](http://www.ensembl.org/Homo_sapiens/searchview?species=;idx=;q=COL16A1) | 29c |
|  | Collagen, type XXI, alpha 1 precursor | [COL21A1](http://www.ensembl.org/Homo_sapiens/searchview?species=;idx=;q=COL21A1) | 29c |
|  | Collagen alpha-1(II) chain precursor | [COL2A1](http://www.ensembl.org/Homo_sapiens/searchview?species=;idx=;q=COL2A1) | 29c |
|  | Collagen alpha-2(VI) chain precursor. | [COL6A2](http://www.ensembl.org/Homo_sapiens/searchview?species=;idx=;q=COL6A2) | 29c |
|  | Collagen, type XXI, alpha 1 precursor | [COL21A1](http://www.ensembl.org/Homo_sapiens/searchview?species=;idx=;q=COL21A1) | 155 |
|  | Collagen alpha-2(IX) chain precursor | [COL92](http://www.ensembl.org/Homo_sapiens/searchview?species=;idx=;q=COL9A2) | 16-1 |

* Experimentally proven
