# Supplementary material for: Common miR-590 Variant rs6971711 Present Only in African Americans Reduces miR-590 Biogenesis
Source: PLoS One. 2016 May 19;11(5):e0156065. doi: 10.1371/journal.pone.0156065 (PMC4873136; doi:10.1371/journal.pone.0156065)
Supplement: S3 Table — (DOCX) [file pone.0156065.s006.docx]

Supplementary Table 3. Distance between selected GWAS cardiac phenotypic markers and variants found in HCM population

| Chromosome Region | GWAS markers | Cardiac phenotype | HCM variants | miR | Distance (kb) |
| --- | --- | --- | --- | --- | --- |
| 13q14.13 | rs958546 | Atrial fibrillation | rs72631826 | miR16-1 | 3789 |
| 20q13.32 | rs6015450 | Blood pressure | rs13040413  rs6122014  ch20:g.61162228 | miR1-1  miR133a-2 | 3411 |
| 1q32.1 | rs7512898 | Electrocardiogra-phic conduction measures | ch1:g.50623143 | miR29c | 150049 |
| 7q31.2 | rs3807989 | Electrocardiogra-phic traits | rs116155675 | miR29b-1 | 14376 |
| 13q14.2 | rs2031532 | Cardiac hypertrophy | rs72631826 | miR16-1 | 542 |
| 13q14.2 | rs157589 | Cardiac hypertrophy | rs72631826 | miR16-1 | 2424 |
| 1q32.2 | rs17259784 | Cardiac hypertrophy | ch1:g.50623143 | miR29c | 158134 |
| 20q13.32 | rs127430 | Cardiovascular disease risk factors | rs13040413  rs6122014  ch20:g.61162228 | miR1-1  miR133a-2 | 3986 |
| 20q13.32 | rs6015450 | Hypertension | rs13040413  rs6122014  ch20:g.61162228 | miR1-1  miR133a-2 | 3393 |
| 20q13.32 | rs16982520 | Hypertension | rs13040413  rs6122014  ch20:g.61162228 | miR1-1  miR133a-2 | 3393 |
| 7q11.23 | rs2286276 | Metabolic traits | rs6971711 | miR590 | 618 |
| 7q11.23 | rs12539316 | Metabolic traits | rs6971711 | miR590 | 627 |
| 1q32.2 | rs2745967 | Resting heart rate | ch1:g.50623143 | miR29c | 157505 |
| 20q13.33 | rs944260 | Sudden cardiac arrest | rs13040413  rs6122014  ch20:g.61162228 | miR1-1  miR133a-2 | 908.35 |
| 18q11.2 | rs16942421 | Sudden cardiac arrest | rs9989532 | miR1-2 | 4747 |
| 20q13.32 | rs6015450 | Systolic blood pressure | rs13040413  rs6122014  ch20:g.61162228 | miR1-1  miR133a-2 | 3400 |
