# Supplementary material for: Common miR-590 Variant rs6971711 Present Only in African Americans Reduces miR-590 Biogenesis
Source: PLoS One. 2016 May 19;11(5):e0156065. doi: 10.1371/journal.pone.0156065 (PMC4873136; doi:10.1371/journal.pone.0156065)
Supplement: S4 Table — (DOCX) [file pone.0156065.s007.docx]

**Supplementary Table 4**. **Comparison of population-based allele frequency between the HCM cohort and ExAc Database**.

| miRNA | SNP | Minor allele | HCM cohort MAF | | ExAc MAF | ExAc MAF |
| --- | --- | --- | --- | --- | --- | --- |
|  |  |  | AA | W | African | European (Finish and non-Finish) |
| miR-590 | rs6971711 | T | 0.082 (9/110) | 0 (0/332) | 0.070 (728/10340) | 0.000 (22/72910) |
| miR-1-2 | rs9989532 | G | 0.091 (2/22) | 0.009 (3/332) | 0.126 (523/4160) | 0.022（353/16082） |
| miR-16-1 | rs72631826 | C | 0.045 (1/22) | 0 (0/332) | 0.021 (213/10222) | 0.000 (1/72878) |
| miR-29b-1 | rs116155675 | G | 0.045 (1/22) | 0 (0/332) | 0.011 (113/10172) | 0.000 (2/72296) |
| miR-133a-2 | rs13040413 | A | 0.273 (6/22) | 0.202 (67/332) | 0.406 (2836/6982) | 0.331 (11800/35630) |

AA, African Americans; W, whites; MAF, minor allele frequency
