# Supplementary material for: Common miR-590 Variant rs6971711 Present Only in African Americans Reduces miR-590 Biogenesis
Source: PLoS One. 2016 May 19;11(5):e0156065. doi: 10.1371/journal.pone.0156065 (PMC4873136; doi:10.1371/journal.pone.0156065)
Supplement: S5 Table — (DOCX) [file pone.0156065.s008.docx]

**Supplementary Table 5**. Primers for miRNA sequencing.

| **miRNAs** | **Forward (5’ to 3’)** | **Reverse (5’ to 3’)** | **Size (bp)** |
| --- | --- | --- | --- |
| miR-1-1 | gagatggattcagggatgga | gctgacacaggcaaagtgac | 490 |
| miR-1-2 | tggtcaaatgcttaggaatgtc | tcactggatcttcttttccttca | 496 |
| miR-133a-1 | cgtacattgatcattccttaatgc | ttgaaatccttaagtcatccataca | 504 |
| miR-133a-2 | agcgagagggtggtgagtt | actttcagggggaagggtct | 505 |
| miR-30c-1 | tgccctgaataaggacatga | gcactggtaggcaagtaccc | 503 |
| miR-30c-2 | agaactgagcaggcagcact | ccagaacttttgtcacttgggta | 505 |
| miR-29a | tgagcatctgtggattttgg | ttcatgatatgctaatagtgaacca | 522 |
| miR-29b-1 | gtgcccttgcctctaaatga | gcagagacctgactgccatt | 500 |
| miR-29b-2 | tttgtagtgactggtgtgaagttaaa | cccccttctctactgtcacct | 501 |
| miR-29c | tgtattgggtgtcgattgtcat | cagcaaaatgcaactagagaaca | 500 |
| miR-23a | gcatgggccctctaggtatc | caaggccagaggaggtgag | 491 |
| miR-21 | ttttgttttgcttgggagga | tttcaaaacccacaatgcag | 515 |
| miR-208a | ccagtggaggaccaagtatga | ttccaccccctccctatatc | 513 |
| miR-208b | gtcctttccaaggccaactc | tagctggagaagcctgagga | 508 |
| miR-195 | aggccatcctggagaagtg | acgagagagggctgaaagg | 506 |
| miR-590 | ttgttttctttcccgcaaac | actgcagttcccaccagaac | 510 |
| miR-15a | ctgggcacagaatggacttc | tgaaaagactatcaataaaactgaaaa | 505 |
| miR-16-1 | attctttaggcgcgaatgtg | aaatgtgcattaaaataaattcctc | 518 |
