# Supplementary material for: Common miR-590 Variant rs6971711 Present Only in African Americans Reduces miR-590 Biogenesis
Source: PLoS One. 2016 May 19;11(5):e0156065. doi: 10.1371/journal.pone.0156065 (PMC4873136; doi:10.1371/journal.pone.0156065)
Supplement: S6 Table — (DOCX) [file pone.0156065.s009.docx]

**Supplementary Table 6**. **DNA and RNA oligo sequences for functional studies**

| **DNA oligos** |  |
| --- | --- |
| pri-miR-590 cloning forward primer | AACTCGAGTTAAAAACTCAGCTTTGGC |
| pri-miR-590 cloning reverse primer | TTGGTACCAAGACGTTAGTATGTGGGCA |
| pri-miR-590 qPCR forward primer | AAACTCAGCTTTGGCTGAACA |
| pri-miR-590 qPCR reverse primer | CATTTCTGACTGGCTACAGGACT |
| GAPDH qPCR forward primer | TCCAAAATCAAGTGGGGCGA |
| GAPDH qPCR reverse primer | AAATGAGCCCCAGCCTTCTC |
| miR-590-5p Northern probe | TGCACTTTTATGAATAAGCTC |
| miR-590-3p-WT Northern probe | ACTAGCTTATACATAAAATTA |
| miR-590-3p-C57T Northern probe | ACTAACTTATACATAAAATTA |
| miR-15-5p Northern probe | CACAAACCATTATGTGCTGCTA |
| miR-16-5p Northern probe | CGCCAATATTTACGTGCTGCTA |
| miR-17-5p Northern probe | CTACCTGCACTGTAAGCACTTTG |
| miR-25-3p Northern probe | TCAGACCGAGACAAGTGCAATG |
| U6 RNA Northern probe | GAATTTGCGTGTCATCCTTGCGCAGGGGCCATGCTAA |
| Forward strand for the miR-590-5p perfect match sites reporter | TCGATGCACTTTTATGAATAAGCTATGCACTTTTATGAATAAGCTATGCACTTTTATGAATAAGCTATGCACTTTTATGAATAAGCTA |
| Reverse strand for the miR-590-5p perfect match sites reporter | GGCCTAGCTTATTCATAAAAGTGCATAGCTTATTCATAAAAGTGCATAGCTTATTCATAAAAGTGCATAGCTTATTCATAAAAGTGCA |
| Forward strand for the miR-590-5p near perfect match sites reporter | TCGATGCACTTTTACCCATAAGCTATGCACTTTTACCCATAAGCTATGCACTTTTACCCATAAGCTATGCACTTTTACCCATAAGCTA |
| Reverse strand for the miR-590-5p near perfect match sites reporter | GGCCTAGCTTATGGGTAAAAGTGCATAGCTTATGGGTAAAAGTGCATAGCTTATGGGTAAAAGTGCATAGCTTATGGGTAAAAGTGCA |
| **RNA oligos** |  |
| pre-miR-590-WT | GAGCUUAUUCAUAAAAGUGCAGUAUGGUGAAGUCAAUCUGUAAUUUUAUGUAUAAGCUAGU |
| pre-miR-590-SNP | GAGCUUAUUCAUAAAAGUGCAGUAUGGUGAAGUCAAUCUGUAAUUUUAUGUAUAAGUUAGU |
| miR-590-5p | GAGCUUAUUCAUAAAAGUGCA |
| miR-590-3p-WT | UAAUUUUAUGUAUAAGCUAGU |
| miR-590-3p-SNP | UAAUUUUAUGUAUAAGUUAGU |
